# Underutilization of albuminuria screening in adults with diabetes mellitus or hypertension: a systematic review and meta-analysis

**DOI:** 10.1186/s12882-025-04672-5

**Published:** 2025-12-04

**Authors:** Mohamed A. Albekery, Ibrahim S. Alhomoud, Lama S. Alabdulathim, Marwah A. Almajed, Amjad A. Alobaid, Manar K. Alomair, Sukainah A. Al Shehab, Khalid A. Alhasan, Abdullah Al Hamid

**Affiliations:** 1 Department of Pharmacy Practice, College of Clinical Pharmacy, King Faisal University, Al-Ahsa, 31982 Saudi Arabia; 2 Department of Pharmacy Practice, College of Pharmacy, Qassim University, Qassim, 51452 Saudi Arabia; 3 Department of Pharmacy, Almoosa Rehabilitation Hospital, Al-Ahsa, 36322 Saudi Arabia; 4 Department of Pharmacy, Obeid Specialized Hospital, Al-Ahsa, 31982 Saudi Arabia; 5 School of Medicine, University College Dublin, Dublin, Ireland; 6 Department of Pediatrics, College of Medicine, King Saud University, Riyadh, Saudi Arabia; 7 Kidney and Pancreas Health Center, Organ Transplant Center of Excellence, King Faisal Specialist Hospital and Research Center, Riyadh, Saudi Arabia

**Keywords:** Albuminuria testing, Chronic kidney disease, Diabetes mellitus, Hypertension, Screening practices, Guideline implementation

## Abstract

**Background:**

Guidelines recommend routine urine albumin-to-creatinine ratio (uACR) screening in patients with diabetes (annual) and hypertension (at least once, with follow-up annually if CKD is found). However, real-world adherence remains uncertain and appears suboptimal. This study aimed to quantify the global prevalence of albuminuria testing in high-risk adults with hypertension and/or diabetes.

**Methods:**

We conducted a systematic review and meta-analysis of observational studies reporting the proportion of adults with diabetes mellitus, hypertension, or both who underwent albuminuria testing in routine care. Multiple databases were searched through Web of Science, PubMed, Google Scholar, and Scopus. Pooled uACR testing prevalence was calculated using random-effects meta-analysis of proportions, with heterogeneity assessed by Cochran’s Q and I² statistics. Subgroup analyses examined differences by patient group, test modality, and country income level.

**Results:**

Thirty studies (from diverse countries and settings) met the inclusion criteria, involving over 29 million patients. Albuminuria testing rates were low in most settings. Overall, only about one in five high-risk patients underwent a uACR test in routine clinical practice. Pooled uACR testing prevalence was approximately 22% (fixed-effect model dominated by a large U.S. dataset), and 49% (95% CI: 7–93%) in a random-effects model accounting for extreme between-study variability (I² ~100%). Testing uptake ranged from < 5% in some systems to nearly 100% in protocolized programs. Subgroup comparisons showed higher rates in diabetes-focused and screening-driven studies, whereas hypertension cohorts had particularly low uptake.

**Conclusions:**

uACR testing remains underutilized among patients with hypertension or diabetes. This gap between guidelines and practice leads to delayed CKD detection and timely initiation of appropriate treatment.

**Clinical trial number:**

Not applicable.

**Supplementary Information:**

The online version contains supplementary material available at 10.1186/s12882-025-04672-5.

## Introduction

Chronic Kidney Disease (CKD) is diagnosed based on reduced estimated glomerular filtration rate (eGFR) and/or elevated albuminuria, both of which independently indicate kidney damage and risk for disease progression and cardiovascular events [1, 2]. Urine albumin-to-creatinine ratio (uACR) testing and eGFR provide complementary information that strengthens the accuracy of CKD diagnosis and risk stratification [3]. In the United States, the National Health and Nutrition Examination Survey (NHANES) estimates that approximately 14% of adults meet the diagnostic criteria for CKD [4]. CKD is estimated to affect approximately 700 million individuals globally [5]. Projections suggest that CKD-related deaths could reach up to 4 million annually by 2040 [6]. Cardiovascular disease and end-stage kidney Disease (ESKD) complications are the leading cause of CKD-related mortality [3].

The asymptomatic nature of early-stage CKD often delays diagnosis [1, 2]. Effective CKD screening protocols involves several critical assessments, including the measurement of serum creatinine, eGFR through a serum creatinine-based equation, evaluation of the uACR, and urinalysis [2]. Urinalysis is often effective in detecting overt proteinuria exceeding 300 mg per 24 h [7]. However, it has limited sensitivity for detecting lower, yet clinically meaningful, levels of albuminuria in the range of 30 to 300 mg per day [7]. Given these limitations, screening for albuminuria should be performed using a spot uACR test, with results interpreted according to established albuminuria categories: normal (< 30 mg/g), moderately increased (30–300 mg/g), and severely increased (>300 mg/g) [2].

uACR testing in high-risk populations is strongly recommended by disease-specific clinical practice guidelines [8–11]. Individuals with hypertension and diabetes mellitus are at the core of the CKD risk population and require early evaluation for CKD using the uACR test [2, 9–11]. Current American Diabetes Association (ADA) and Kidney Disease: Improving Global Outcomes (KDIGO) guidelines recommend annual uACR testing for adults with type 2 diabetes, and initiation of testing 5 years after diagnosis in those with type 1 diabetes [9]. For individuals with hypertension, the 2025 AHA/ACC Hypertension Guideline recommends performing uACR testing at least annually for all patients to evaluate the development or progression of kidney disease [10]. These populations are particularly at significant risk because both conditions accelerate glomerular injury, endothelial dysfunction, and systemic inflammation, leading to a markedly higher risk of kidney failure, cardiovascular events, and mortality when albuminuria is present [8–11].

Current evidence suggests that the real-world utilization of uACR testing remains substantially below guideline recommendations. Global real-world data from an individual participant data meta-analysis of over 3.6 million adults across 44 countries indicate that only about one in every three patients with diabetes (35.1%; range across cohorts: 12.3%–74.5%) and approximately one in every 25 patients with hypertension (4.1%; range: 1.3%–20.7%) undergo uACR testing [12]. Similarly, analysis of a nationally representative U.S. cohort of adults with hypertension and/or diabetes mellitus revealed that only 17.5% underwent uACR testing, suggesting that a substantial proportion of albuminuria cases may remain undetected [13].

To address the ongoing gap between clinical guidelines and real-world practice, we conducted a comprehensive meta-analysis to evaluate the prevalence of uACR testing among individuals with hypertension and/or diabetes mellitus. By systematically integrating data from diverse healthcare settings and patient populations, this study aims to provide the first global estimate of real-world uACR testing rates. The findings establish a data-driven foundation for improving early CKD detection and reducing the burden of kidney and cardiovascular diseases in high-risk groups.

## Methods

### Protocol and reporting

This review was conducted in accordance with the Preferred Reporting Items for Systematic Reviews and Meta-Analyses (PRISMA) guidelines [14].

### Eligibility criteria

We included observational studies reporting the prevalence of albuminuria and proteinuria testing among adults with diabetes mellitus, hypertension, or both. Studies were eligible if they reported the proportion of participants who received at least one albuminuria assessment during the study period. For inclusion in the meta-analysis, studies were required to provide extractable numerators and denominators for routine-care uACR testing prevalence. Studies that lacked compatible prevalence data, such as those reporting only implementation outcomes or descriptive commentary, were included in the narrative synthesis but excluded from quantitative pooling.

### Information sources and search strategy

We systematically searched four electronic databases, including PubMed, Scopus, Web of Science, and Google Scholar, from inception to January 2024. A comprehensive Boolean search strategy was developed using controlled vocabulary and free-text terms related to uACR testing, proteinuria, chronic kidney disease, diabetes, and hypertension (such as (“albuminuria” OR “proteinuria” OR “urinary albumin excretion” OR “microalbuminuria”) AND (“chronic kidney disease” OR “CKD” OR “diabetes mellitus” OR “hypertension” OR “high blood pressure”) AND (“screening” OR “testing” OR “prevalence”)). A complete example of the PubMed search strategy is provided in Supplementary File 1.

### Study selection

All retrieved records were imported into *EndNote 21*, and duplicates were removed. Five reviewers (*LA/ MA/ MO/ AO/ SS*) independently screened titles and abstracts, followed by full-text review to confirm eligibility. Disagreements were resolved through discussion or adjudication by third reviewers (*MAB/ AAH*).

### Data extraction and management

Data were extracted using a structured template capturing: study author, year, country, design, population characteristics, sample size, care setting, assay type, number tested, and uACR testing prevalence. Harmonization involved collapsing variations of “primary care” into a single category for subgroup analysis. Any discrepancies during data entry were resolved by discussion.

### Risk of bias assessment

Quality checks were rigorously implemented by assessing the risk of bias of the included studies using the Joanna Briggs Institute (JBI) Critical Appraisal Checklist for Studies Reporting Prevalence Data [15]. This tool evaluates representativeness of the sampling frame, adequacy of sample size, reliability of outcome measurement, appropriateness of statistical analysis, and handling of response rates. Each study was assigned an overall rating of low, moderate, or high risk based on domain-level judgments. Summaries of the item-level and domain-level ratings are presented in Fig. 2 (traffic-light plot) and 3 (domain-level summary plot). The full item-level scoring matrix for all included studies is provided in Supplementary File 2.

### Statistical analysis

We performed meta-analysis in R (version 4.4.0) using the meta package. Study-level proportions of uACR testing were logit-transformed, and pooled estimates were computed using the Hartung–Knapp random-effects model with restricted maximum likelihood (REML) estimation for between-study variance. Fixed-effect estimates were calculated for comparison. Between-study heterogeneity was quantified with Cochran’s Q statistic, I², τ², and 95% prediction intervals.

Sensitivity analyses evaluated the impact of (i) excluding studies rated high risk of bias, (ii) omitting large datasets (> 1,000,000 participants), and (iii) including studies where testing was protocol-driven. Subgroup analyses explored differences by patient population (diabetes only, hypertension only, combined), assay type (dipstick vs. quantitative), and country income level. Publication bias was assessed using funnel plots and Egger’s regression test, recognizing limited power due to small numbers of pooled studies.

Forest plots summarizing individual and pooled prevalence estimates were generated using the forest() function in the meta package. All analyses were reproducible through a single R script; no additional external datasets were used.

## Results

### Search and study selection

**Fig. 1:**
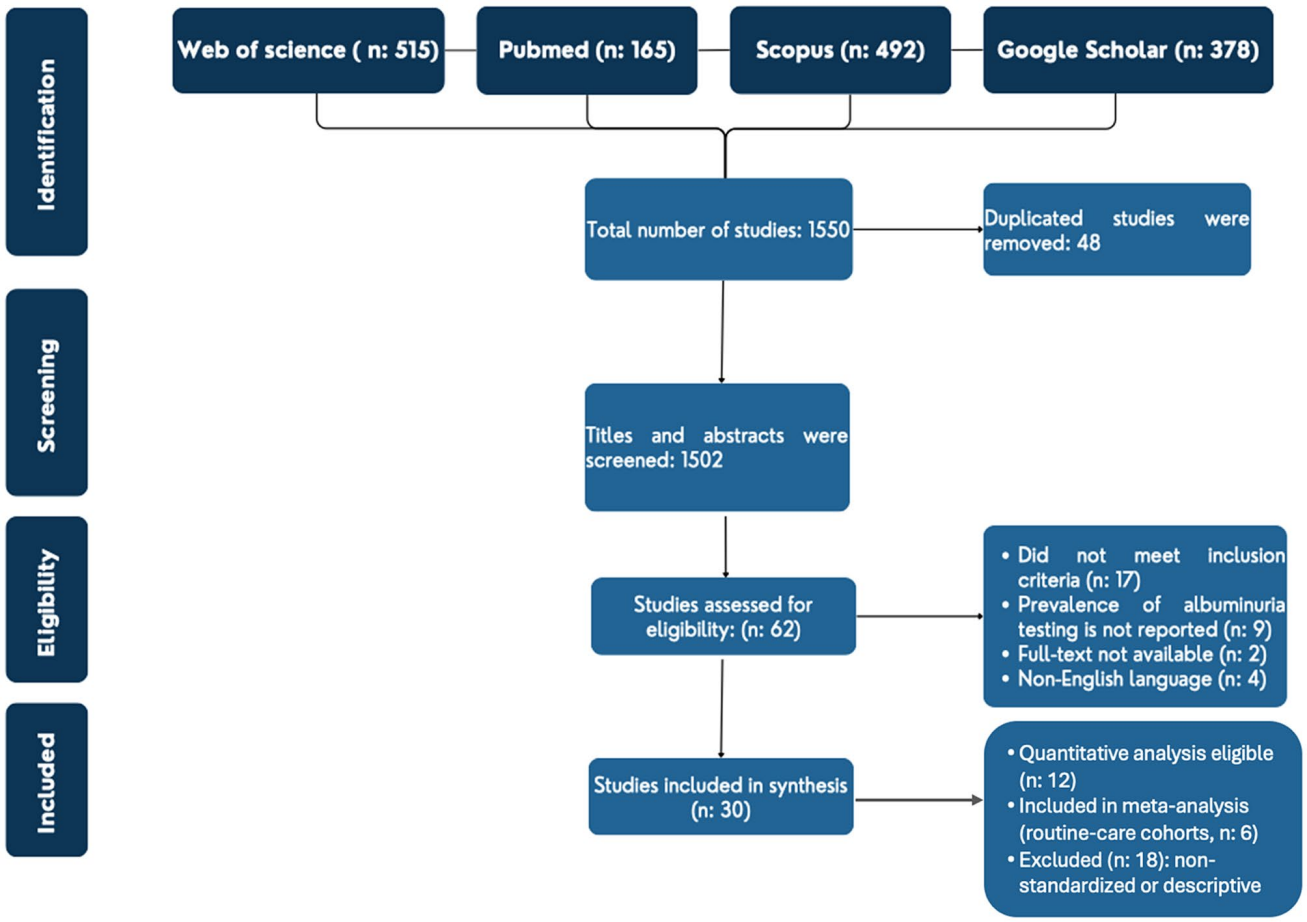
PRISMA flow diagram of study selection. Of 30 included studies, 12 had extractable numerators and denominators, and 6 routine-care cohorts were included in the quantitative meta-analysis. The remaining 18 studies were excluded from quantitative pooling due to descriptive design, mixed denominators, or non-standardized prevalence data

A systematic search of PubMed, Scopus, Web of Science, and Google Scholar identified 1550 records. After removing 48 duplicates, 1,502 titles and abstracts were screened, of which 1,440 were excluded. The remaining 62 full-text articles were assessed for eligibility, and 30 studies met the inclusion criteria. (Fig. 1). Of these, only 12 studies reported extractable numerator and denominator pairs for routine-care uACR testing, enabling proportion-based meta-analysis. The remaining 18 lacked standardized uACR testing prevalence data, presented mixed denominators, or were descriptive; these were unsuitable for quantitative pooling but informed the narrative synthesis.

### Study characteristics summary

The 30 included investigations encompass a broad range of settings and methodologies. Geographically, seven were conducted in high-income countries—the United States; United Kingdom; Australia; Finland; Taiwan; Portugal; and Korea—and ten originated from middle-income contexts including India; Indonesia; Cuba; Egypt; Guatemala; Kenya; Moldova; Malaysia; Peru; and Serbia. Study designs are divided almost evenly between cohort analyses of routine-care uACR testing uptake over time [12, 13, 16–27] and cross-sectional audits or surveys measuring single-time-point uACR testing prevalence [28–43].

Sample sizes varied dramatically—from as few as 72 participants in a Finnish 24-hour albumin excretion study [28] to over 28 million individuals in a U.S. laboratory database assessment [16, 28]. Populations studied included those with diabetes mellitus, hypertension, both conditions, undiagnosed hypertensives, and first-degree relatives of CKD patients. Testing modalities ranged from dipstick urinalysis in early audits to quantitative ACR assays in electronic health records and laboratory datasets [13, 27]. Care settings spanned primary-care chart reviews, specialty outpatient clinics, multi-site electronic health record (EHR) systems, national lab databases, and community screening programs.

Reported uACR testing prevalence exhibited extreme variability, ranging from as low as 4.5% to forced-protocol uptake of 100% in several cohorts [21, 28, 34, 42]. This reflects the spectrum from routine practice gaps to research-driven saturation. Detailed characteristics of all included studies, including design, population, assay type, care setting, and reported uACR testing prevalence, are summarized in Table 1.

**Table 1:** Studies characteristics

Study ID	Country	Design	Sample Size	Population	Assay Type	Care Setting	Risk-of-Bias	Testing Prevalence (%)
Guthrie & Lott (1993) [21]	USA	Cohort	796	DM & HTN	Dipstick	Primary-care audit	Moderate	100
Kissmeyer et al. (1999) [25]	UK	Cohort	2 561	HTN only	Dipstick	Primary-care audit	Moderate	29
McCullough et al. (2008) [24]	USA	Cohort	86 305	DM & HTN	uACR & eGFR	Multi-site EHR	Moderate	KEEP: 86.6% NHANES: 83%
Hitha et al. (2008) [26]	India	Cohort	150	HTN only	ACR strip & Micral strip	Single-site clinic	High	100
Herrera Valdés et al. (2010) [22]	Cuba	Cohort	2 762	DM & HTN	uACR & Dipstick	Population survey	Low	100
Ghafari et al. (2010) [20]	Iran	Cohort	905	DM & HTN	Dipstick	Community screening	Moderate	100
Codreanu et al. (2012) [17]	Moldova	Cohort	1 025	DM & HTN	Dipstick	Population survey	Low	100
Major et al. (2015) [23]	UK (South Asians)	Cohort	6 082	Undiagnosed HTN; Normotensiv; Known HTN	uACR strip	Primary-care based	Low	90.11
Flood et al. (2018) [19]	Guatemala	Cohort	144	T2DM	Dipstick & uACR	Programmatic screening	Moderate	100
Dash et al. (2018) [18]	India	Cohort	6 175	DM & HTN	Dipstick & uACR	Nephrology OPD	Moderate	45.6
Shin et al. (2021) [12]	Global	Cohort	1 303 027	DM & HTN	ACR	Multi-cohort consortium	Moderate	DM 35.1; HTN 4.1
Alfego et al. (2021) [16]	USA	Cohort	28 295 982	DM & HTN	uACR ± uPCR	LabCorp database	Low	21
Stempniewicz et al. (2021) [27]	USA	Cohort	513 165	T2DM	eGFR & uACR	Multi-org EHR	Low	53
Chu et al. (2023) [13]	USA & UK	Cohort	192 108	DM & HTN	uACR	Multi-state EHR	Low	DM 52.3; HTN 5.1
Forsblom et al. (1992) [28]	Finland	Cross-sec	72	T1DM	24-h AER	Specialty clinic	Moderate	77.7
Thomas (2008) [42]	Australia	Cross-sec	3 893	T2DM	Dipstick & Micral strip	GP clinics	Moderate	100
Król et al. (2009) [29]	Poland	Cross-sec	2 471	DM & HTN	Dipstick	Population screening	Low	25.4
Soegondo et al. (2009) [30]	Indonesia	Cross-sec	770	T2DM	ACR strip	Clinic-based	Low	80
van der Meer et al. (2010) [31]	Netherlands	Cross-sec	1 291	DM & HTN	uACR	GP practices	Low	T2DM 97; HTN 87
Gouda et al. (2011) [32]	Egypt	Cross-sec	683	Relatives of CKD	Dipstick	Family screening	Moderate	61
Chiang et al. (2011) [33]	Taiwan	Cross-sec	1 827	T2DM	ACR strip	Clinic	Low	70.5
Ležaić et al. (2011) [34]	Serbia	Cross-sec	1 617	DM & HTN	Dipstick & ACR	Population survey	Low	4.5
Medina et al. (2012) [43]	Peru	Cross-sec	2 968	DM & HTN	Dipstick & ACR	Nephrology OPD	Moderate	53.45
Ong et al. (2013) [35]	Malaysia	Cross-sec	46 961	DM & HTN	Dipstick	Health screening	Moderate	100
Polónia et al. (2016) [36]	Portugal	Cross-sec	9 198	HTN ± T2DM	24-h AER & Micral strip	Clinic	Low	33.3
Kang et al. (2016) [37]	Korea	Cross-sec	6 045	DM & HTN	Dipstick & ACR	Registry data	Low	97.7
Wong et al. (2017) [40]	USA (HI)	Cross-sec	1 190	DM & HTN	uACR & spot albumin	Community screening	Moderate	98.9
Burrows et al. (2018) [38]	USA	Cross-sec	894	DM & HTN	Dipstick	Pilot screening	Low	53.9
Lee et al. (2019) [39]	USA	Cross-sec	16 414	HTN ± DM	uACR	Safety-net primary care	Low	35
Otieno et al. (2020) [41]	Kenya	Cross-sec	723	T2DM	Dipstick & ACR	Hospital clinic	Low	53

Abbreviations: ACR = Albumin-to-Creatinine Ratio; AER = Albumin Excretion Rate; CKD = Chronic Kidney Disease; Cross-sec = Cross-sectional; DM = Diabetes Mellitus; EHR = Electronic Health Record; eGFR = Estimated Glomerular Filtration Rate; GP = General Practice; HI = Hawaii; HTN = Hypertension; OPD = Outpatient Department; T1DM = Type 1 Diabetes Mellitus; T2DM = Type 2 Diabetes Mellitus; uACR = Urinary Albumin-to-Creatinine Ratio; UPCR = Urinary Protein-to-Creatinine Ratio

### Risk of bias summary

**Fig. 2:**
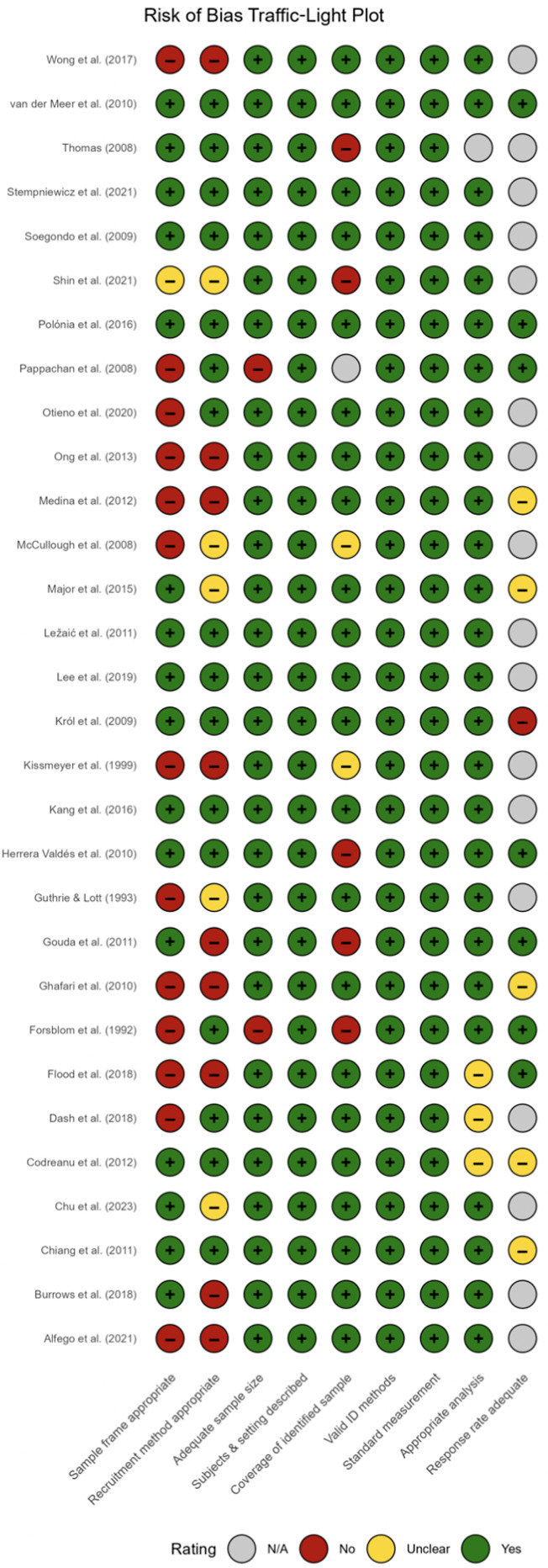
Risk of bias traffic plot

**Fig. 3:**
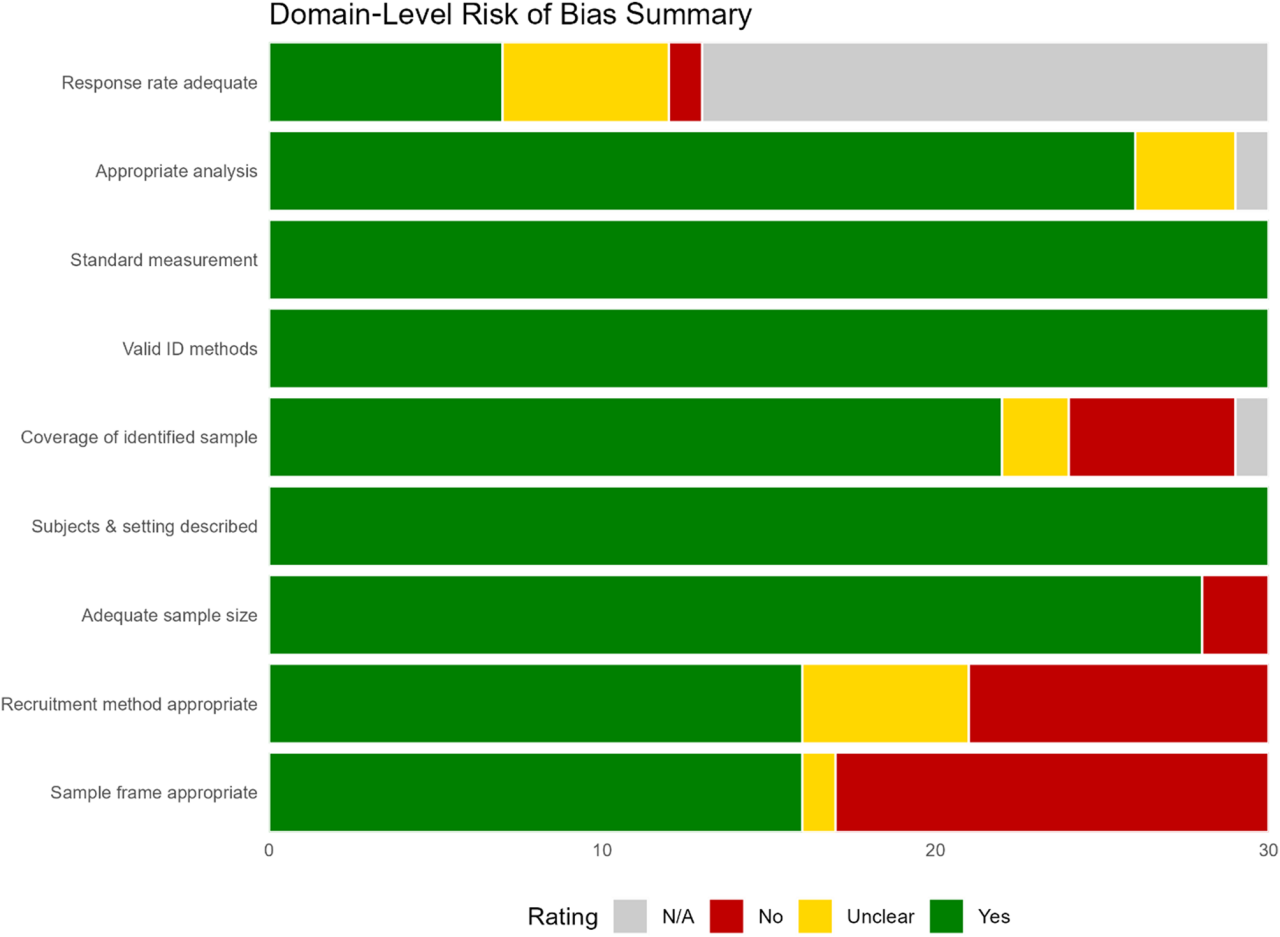
Risk of bias summary plot

Risk of bias was evaluated using the Joanna Briggs Institute checklist for prevalence studies, which covers nine domains from sample frame to response rate [15]. Across the 30 included studies, 16 were judged at low risk, 13 at moderate risk, and one at high risk [26]. The study categorized as high risk relied on a small (*n* = 150), clinic-based sample with no clear sampling frame and unbalanced measurement protocols, warranting its sole high-risk classification [26]. The most common shortcomings were use of non-representative or convenience samples, reliance on dipstick-only uACR testing without confirmatory assays, and failure to report or manage response rates. Together, these issues highlight potential limitations in generalizability, measurement validity, and attrition bias. A full domain-level breakdown is shown in Fig. 2 and 3, which visually summarize each study’s judgments across the nine domains.

### Quantitative synthesis

#### Primary random-effects model

**Fig. 4:**
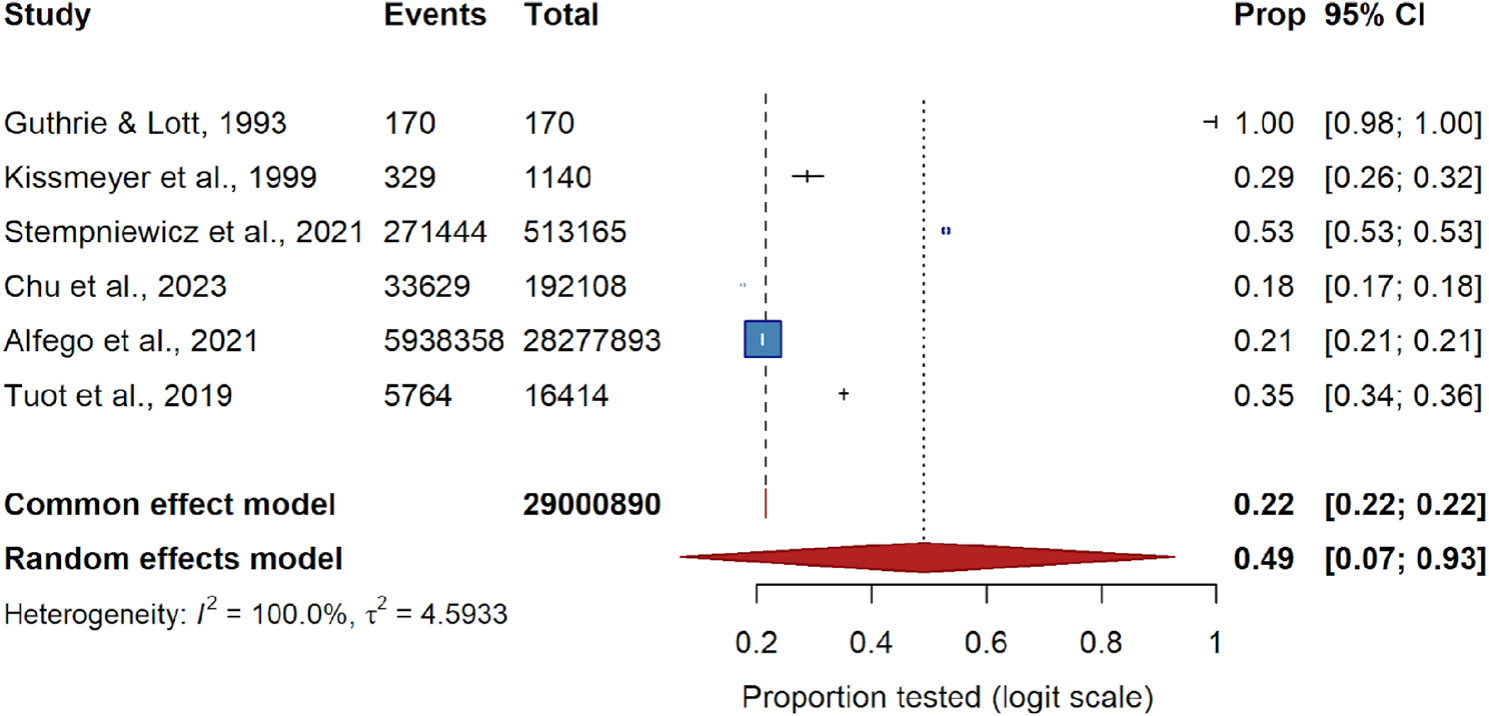
Pooled primary prevalence

The six routine-care cohorts collectively included 29,008,890 at-risk adults [13, 16, 21, 25, 27, 39]. On the logit scale, the Hartung–Knapp random-effects model estimated a pooled uACR testing prevalence of 49% (95% CI 7–93%), while the fixed-effect model, heavily influenced by the LabCorp dataset, produced a prevalence of 22% (Fig. 4). Between-study heterogeneity was extreme (I² = 100%, τ² = 4.59), and the 95% prediction interval (0.7–63.4%) indicated that uptake can be negligible in some health systems and exceed 60% in others. This degree of heterogeneity suggests that variation in uACR testing rates reflects true differences in clinical implementation across health systems rather than random variation. When the large U.S. LabCorp dataset (>28 million participants) was excluded in sensitivity analysis, the pooled prevalence estimate remained comparable. Sensitivity analyses incorporating additional cohorts are presented below.

#### Sensitivity analyses

The six routine-care cohorts (Guthrie and Lott 1993; Kissmeyer et al. 1999; Stempniewicz et al. 2021; Chu et al. 2023; Alfego et al. 2021; Tuot et al. 2019) collectively included 29,008,890 at-risk adults. On the logit scale, the Hartung–Knapp random-effects model estimated a pooled testing prevalence of 49% (95% CI 7–93%), while the fixed-effect model, heavily influenced by the LabCorp dataset, produced a prevalence of 22%. Between-study heterogeneity was extreme (I² = 100%, τ² = 4.59), and the 95% prediction interval (0.7–63.4%) indicated that uptake can be negligible in some health systems and exceed 60% in others. Sensitivity analyses incorporating additional cohorts are presented.

#### Sub-group effects

**Fig. 5:**
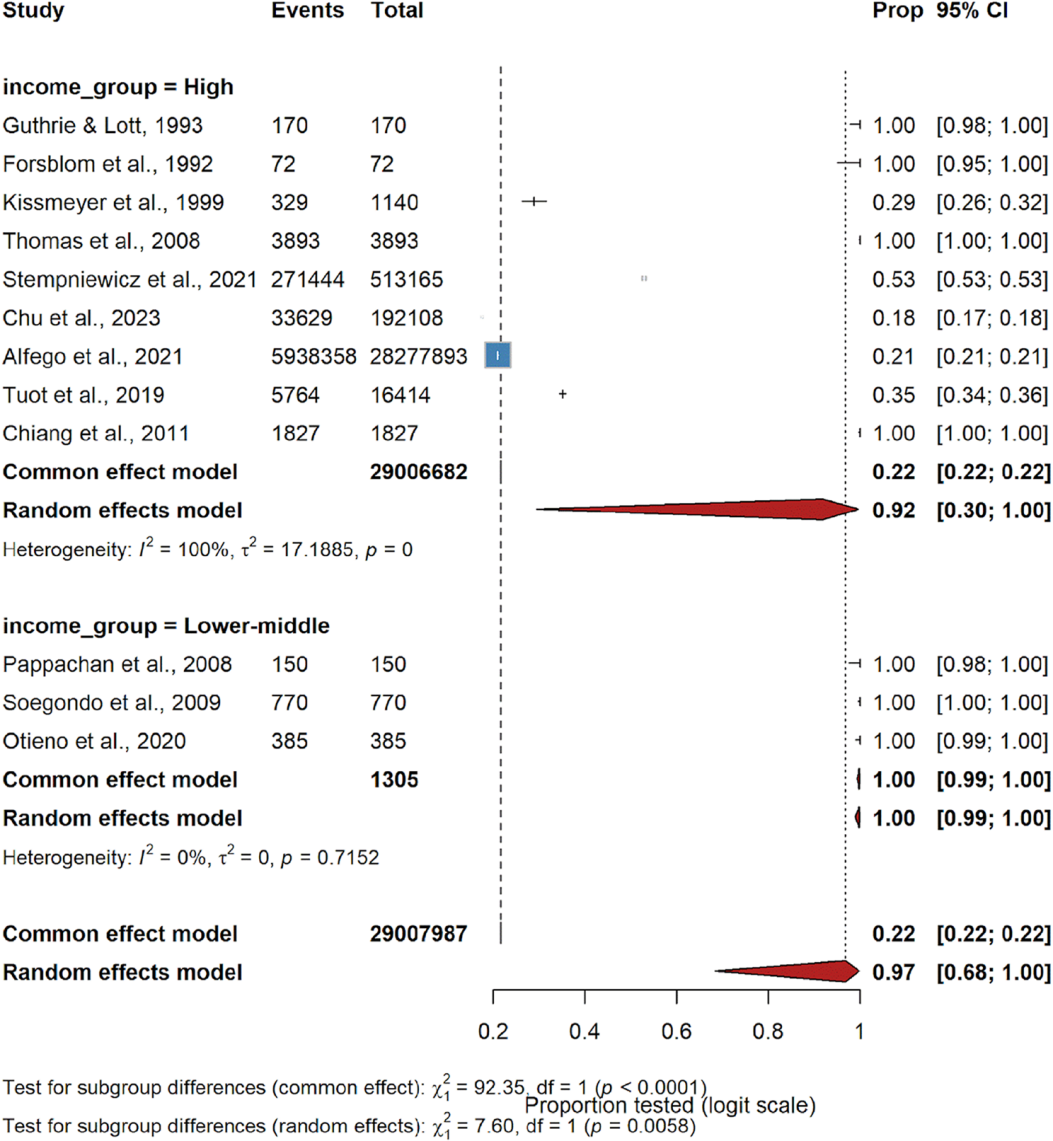
Income subgroup

Within routine-care cohorts, quantitative uACR testing averages 35% (17–60%), whereas dipstick audits appear to reach 89% (33–99%). Diabetes-only cohorts approach universal coverage (99%), hypertension-only cohorts reach 88% and mixed diabetes–hypertension samples remain at 51% (*χ*² = 9.98, *df* = 4, *p* = 0.041). Income stratification mirrors this gradient: high-income research environments cluster at 92%, while externally funded lower-middle-income screens report 100% (*χ*² = 7.60, *df* = 1, *p* = 0.006; Fig. 5). Healthcare settings may also influence the extent of uACR testing uptake. Forest plots for the subgroup analyses are provided in the Supplementary Materials (Supplement 3 and Supplement 4).

#### Model diagnostics

**Fig. 6:**
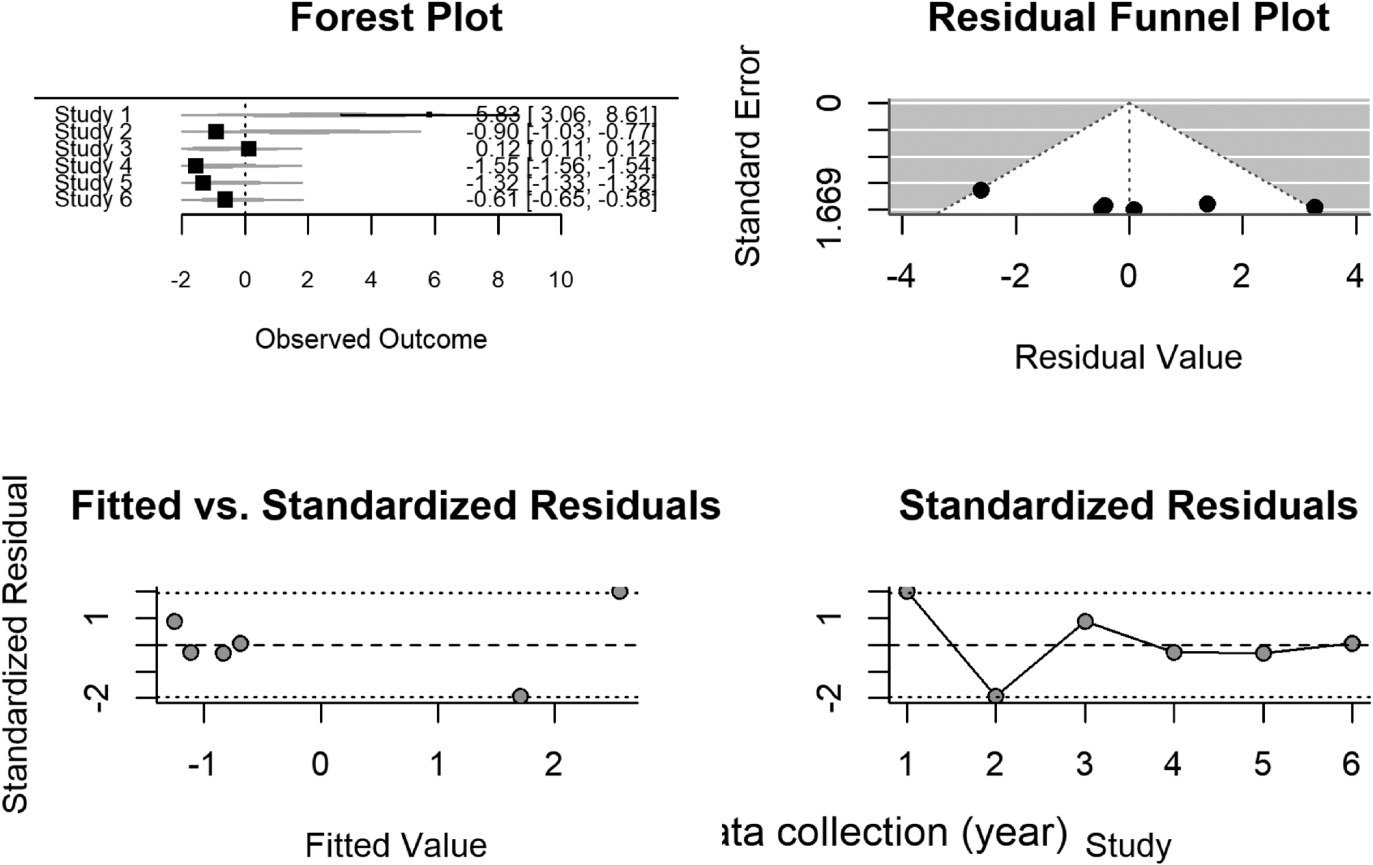
Test for publication bias

Visual inspection of funnel plots and Q–Q residual plots (Fig. 6) indicated no strong evidence of small-study effects, though formal Egger tests were underpowered (k = 6). Standardized residuals fell within ± 2.5, suggesting an adequate overall model fit despite substantial heterogeneity.

### Narrative analysis

There are several revealed reasons for the stalling of guideline-driven uACR testing in practice. Studies point to knowledge or attitudinal gaps among frontline clinicians such as limited familiarity with guideline-recommended screening targets in busy primary-care settings [17, 18, 20, 22, 29, 31, 37, 40, 43]. Additional barriers include supply-chain disruptions and cost-related constraints [32]. In some settings, periodic shortages of reagent strips have led to restricted access, with testing limited to symptomatic patients when resources are scarce [17, 20, 29, 41]. The cost of uACR testing supplies has also been reported to exceed local healthcare budgets [26], and even where laboratory capacity exists, out-of-pocket charges may deter patient access to testing [23, 36, 38, 39, 42].

These barriers to uACR testing have meaningful clinical consequences. In Cuba, half of adults who tested positive for uACR also had uncontrolled hypertension but were not receiving renin–angiotensin system blockers [22]. In rural Guatemala, a screening initiative found that one-third of positive tests revealed previously unrecognized cardiovascular risk factors [19]. In contrast, the Dutch SALINE trial demonstrated a scalable solution: mailing quantitative uACR testing kits to high-risk individuals resulted in a 40% participation rate, identified previously undetected albuminuria in one out of every twelve participants, and prompted therapy intensification in nearly half of those newly diagnosed [44]. Taken together, this evidence aligns with the quantitative signal, indicating that low uACR testing prevalence is driven less by test performance than by modifiable deficits in clinician awareness, supply-chain resilience, and patient affordability.

## Discussion

### Principal findings

Despite three decades of guideline exhortation, our meta-analysis shows that only one in five adults with diabetes or hypertension in routine care actually receives a quantitative uACR test. Fixed-effect pooling, dominated by a 28-million-record laboratory denominator, yields an even starker figure of 22%; the random-effects model, which cushions extreme heterogeneity, rises to 49% but still falls far short of universal coverage. A 95% prediction interval reaching down to < 1% confirms that, in some health-care systems, uACR testing is virtually absent.

The common-effect model was largely influenced by the large U.S. dataset from Alfego et al. (2021), and the extreme between-study heterogeneity (I² ≈ 100%) produced a wide prediction interval, indicating that the pooled prevalence should be interpreted with caution. The pronounced heterogeneity observed across studies underscores that uACR testing uptake is highly context dependent, shaped by variations in healthcare infrastructure, clinical practices, reimbursement frameworks, and laboratory capacity. Given that the included studies differed in duration, data source, and testing protocol, the pooled prevalence should be interpreted as a global signal of underutilization—an index of opportunity for system-level improvement—rather than a uniform rate generalizable to all clinical environments.

### Comparison with the contemporary literature

Our estimates dovetail with the largest individual-participant analysis to date, which documented annual uACR testing in 35% of people with diabetes and a mere 4% of those with hypertension across 13 countries [12]. Current guidelines recommend annual measurement of eGFR and uACR in adults with diabetes and at least one-time testing in those with hypertension, with annual follow-up if CKD is confirmed [2, 9–11, 45]. Even in highly resourced health systems in the United States, a joint ADA–KDIGO audit confirms that <50% of adults with type 2 diabetes undergo guideline-concordant uACR testing [9]. These external data triangulate our finding that the global implementation gap is not a statistical artefact but a persistent reality.

### Mechanistic and therapeutic implications

Failure to perform uACR testing is not without consequence, as it may delay the initiation of guideline-recommended therapies. In a global analysis, undiagnosed albuminuria was 20-times more prevalent than detected cases among adults with hypertension, and a positive uACR result was associated with a triple increase in the initiation of renin–angiotensin–aldosterone system (RAAS) blockade [12]. Early diagnosis and initiation of an angiotensin-converting enzyme (ACE) inhibitor or angiotensin II receptor blocker (ARB) has been shown to slow disease progression and improve cardiovascular outcomes [46–48]. Additionally, sodium–glucose cotransporter 2 (SGLT2) inhibitors, glucagon-like peptide-1 (GLP-1) receptor agonists, and finerenone have demonstrated further clinical benefits when added to RAAS blockade, depending on patient-specific appropriateness [48–50]. Inadequate uACR testing consequently eliminates a clinically actionable opportunity to initiate interventions that may attenuate the progression of kidney and cardiovascular disease.

### Implementation strategies

Descriptive evidence from the 18 narrative-only studies highlights three modifiable bottlenecks: limited clinician awareness of annual testing targets, reagent shortages or high per-strip costs, and patient co-payment barriers. The Dutch SALINE trial offers a proof-of-concept model in which mailing quantitative uACR kits to high-risk adults achieved 40% participation, uncovered silent albuminuria in 8.7%, and prompted therapy escalation in 44% of new positive cases [44]. Embedding automatic uACR ordering into electronic health record templates, subsidizing quantitative assays, and publicly reporting adherence against quality benchmarks are actionable strategies that could help close the testing gap and improve early detection efforts. Among these strategies, automated EHR-based test ordering appears to be the most pragmatic and scalable approach across diverse health care settings.

### Strengths and limitations

The strengths of our analysis include assembling the largest pooled denominator to date (~ 29 million) by integrating data from diverse populations, care settings, and income regions, extending insights beyond those from single large national datasets. Additional strengths include rigorous Hartung–Knapp inference and structured subgroup analyses by disease type and country income level. Limitations are residual heterogeneity, underpowered small-study bias tests and reliance on administrative coding in two mega-datasets. Furthermore, most data from lower- and middle-income countries originate from externally funded, short-term screening initiatives rather than established clinical programs. These studies may overestimate the true rate of uACR testing prevalence in routine care and highlight the need for sustainable, locally integrated screening initiatives.

## Conclusion

Routine uACR testing stands as the most neglected yet critical step in CKD prevention. Mandates from ADA, KDIGO and European cardiovascular societies are unanimous, yet real-world uptake languishes well below 50%. The considerable heterogeneity observed across studies reflects genuine differences in healthcare infrastructure, clinical priorities, and implementation capacity rather than inconsistency in evidence. Bridging this gap will require coordinated efforts to enhance clinician awareness, reduce assay costs, and improve patient access.

## Electronic Supplementary Material

Below is the link to the electronic supplementary material.

Supplementary Material 1

Supplementary Material 2

Supplementary Material 3

Supplementary Material 4

## Data Availability

All data generated or analyzed during this study are included in this published article and its supplementary information files.

## References

[1] Webster AC, Nagler EV, Morton RL, Masson P. Chronic kidney disease. Lancet. 2017;389(10075):1238–52. 10.1016/S0140-6736(16)32064-5. 27887750 10.1016/S0140-6736(16)32064-5

[2] Stevens PE, Ahmed SB, Carrero JJ, et al. KDIGO 2024 clinical practice guideline for the evaluation and management of chronic kidney disease. Kidney Int. 2024;105(4):S117–314. 10.1016/j.kint.2023.10.018. 38490803 10.1016/j.kint.2023.10.018

[3] Association of estimated glomerular filtration rate. And albuminuria with all-cause and cardiovascular mortality in general population cohorts: a collaborative meta-analysis. Lancet. 2010;375(9731):2073–81. 10.1016/S0140-6736(10)60674-5. 20483451 10.1016/S0140-6736(10)60674-5 PMC3993088

[4] United States Renal Data System. 2024 USRDS Annual Data Report: Epidemiology of Kidney Disease in the United States. National Institutes of Health, National Institute of Diabetes and Digestive and Kidney Diseases, Bethesda, MD, 2024.

[5] Francis A, Harhay MN, Ong ACM, et al. Chronic kidney disease and the global public health agenda: an international consensus. Nat Rev Nephrol. 2024;20(7):473–85. 10.1038/s41581-024-00820-6. 38570631 10.1038/s41581-024-00820-6

[6] Bikbov B, Purcell CA, Levey AS, et al. Global, regional, and National burden of chronic kidney disease, 1990–2017: a systematic analysis for the global burden of disease study 2017. Lancet. 2020;395(10225):709–33. 10.1016/S0140-6736(20)30045-3. 32061315 10.1016/S0140-6736(20)30045-3 PMC7049905

[7] Gaitonde DY, Cook DL, Rivera IM. Chronic kidney disease: detection and evaluation. Am Fam Physician. 2017;96(12):776–83. 29431364

[8] American Diabetes Association Professional Practice Committee. American Diabetes Association Professional Practice Committee. 4. Comprehensive medical evaluation and assessment of comorbidities: standards of care in diabetes-2025. Diabetes Care. 2025;48(Supplement_1):S59-S85. 10.2337/dc25-S004. 10.2337/dc25-S004 PMC11635044 39651988

[9] de Boer IH, Khunti K, Sadusky T, et al. Diabetes management in chronic kidney disease: A consensus report by the American diabetes association (ADA) and kidney disease: improving global outcomes (KDIGO). Diabetes Care. 2022;45(12):3075–90. 10.2337/dci22-0027. 36189689 10.2337/dci22-0027 PMC9870667

[10] Writing Committee Members*, Jones DW, Ferdinand KC, Taler SJ, Johnson HM, Shimbo D, AHA/ACC/AANP/AAPA/ABC/ACCP/ACPM/AGS/AMA/ASPC et al. /NMA/PCNA/SGIM Guideline for the prevention, detection, evaluation and management of high blood pressure in adults: a report of the American college of cardiology/American Heart association joint committee on clinical practice guidelines. Hypertens Dallas Tex 1979. 2025;82(10):e212–316. 10.1161/HYP.0000000000000249 40811516

[11] McEvoy JW, McCarthy CP, Bruno RM, et al. 2024 ESC guidelines for the management of elevated blood pressure and hypertension. Eur Heart J. 2024;45(38):3912–4018. 10.1093/eurheartj/ehae178. 39210715 10.1093/eurheartj/ehae178

[12] Shin JI, Chang AR, Grams ME, et al. Albuminuria testing in hypertension and diabetes: an Individual-Participant data Meta-Analysis in a global consortium. Hypertension. 2021;78(4):1042–52. 10.1161/HYPERTENSIONAHA.121.17323. 34365812 10.1161/HYPERTENSIONAHA.121.17323 PMC8429211

[13] Chu CD, Xia F, Du Y, et al. Estimated prevalence and testing for albuminuria in US adults at risk for chronic kidney disease. JAMA Netw Open. 2023;6(7):e2326230. 10.1001/jamanetworkopen.2023.26230. 37498594 10.1001/jamanetworkopen.2023.26230 PMC10375308

[14] Page MJ, McKenzie JE, Bossuyt PM, et al. The PRISMA 2020 statement: an updated guideline for reporting systematic reviews. BMJ. 2021;372:n71. 10.1136/bmj.n71. 33782057 10.1136/bmj.n71 PMC8005924

[15] Munn Z, Moola S, Lisy K, Riitano D, Tufanaru C. Methodological guidance for systematic reviews of observational epidemiological studies reporting prevalence and cumulative incidence data. Int J Evid Based Healthc. 2015;13(3):147–53. 10.1097/XEB.0000000000000054. 26317388 10.1097/XEB.0000000000000054

[16] Alfego D, Ennis J, Gillespie B, et al. Chronic kidney disease testing among At-Risk adults in the U.S. Remains low: Real-World evidence from a National laboratory database. Diabetes Care. 2021;44(9):2025–32. 10.2337/dc21-0723. 34353883 10.2337/dc21-0723 PMC8740927

[17] Codreanu I, Sali V, Gaibu S, et al. Prevalence of hypertension and diabetes and coexistence of chronic kidney disease and cardiovascular risk in the population of the Republic of Moldova. Int J Hypertens. 2012;2012:1–8. 10.1155/2012/951734. 10.1155/2012/951734 PMC3515913 23251790

[18] Dash SC, Agarwal SK, Panigrahi A, Mishra J, Dash D. Diabetes, hypertension and kidney disease combination DHKD syndrome is common in India. J Assoc Physicians India. 2018;66(3):30–3. 30341865

[19] Flood D, Garcia P, Douglas K, Hawkins J, Rohloff P. Screening for chronic kidney disease in a community-based diabetes cohort in rural guatemala: a cross-sectional study. BMJ Open. 2018;8(1):e019778. 10.1136/bmjopen-2017-019778. 29358450 10.1136/bmjopen-2017-019778 PMC5781190

[20] Ghafari A, Ahmadnezhad E, Sepehrvand N, et al. Screening for asymptomatic kidney disease in high-risk population of Urmia, Iran. Iran J Kidney Dis. 2010;4(4):307–11. 20852372

[21] Guthrie RM, Lott JA. Screening for proteinuria in patients with hypertension or diabetes mellitus. J Fam Pract. 1993;37(3):253–6. 8409876

[22] Herrera R, Almaguer M, Chipi J, et al. Albuminuria as a marker of kidney and cardio-cerebral vascular damage. Isle of youth study (ISYS), Cuba. MEDICC Rev. 2010;12(4):20–6. 10.37757/MR2010.V12.N4.5. 21048540 10.37757/MR2010.V12.N4.5

[23] Major R, Davies M, Crasto W, Gray L, Webb D, Khunti K. Association between undiagnosed hypertension and microalbuminuria in South Asians without known diabetes. J Hum Hypertens. 2015;29(3):185–9. 10.1038/jhh.2014.62. 25119886 10.1038/jhh.2014.62

[24] McCullough PA, Li S, Jurkovitz CT, et al. CKD and cardiovascular disease in screened High-Risk volunteer and general populations: the kidney early evaluation program (KEEP) and National health and nutrition examination survey (NHANES) 1999–2004. Am J Kidney Dis. 2008;51(4):S38–45. 10.1053/j.ajkd.2007.12.017. 18359407 10.1053/j.ajkd.2007.12.017

[25] Kissmeyer L, Kong C, Cohen J, Unwin RJ, Woolfson RG, Neild GH. Community nephrology: audit of screening for renal insufficiency in a high risk population. Nephrol Dial Transpl Off Publ Eur Dial Transpl Assoc - Eur Ren Assoc. 1999;14(9):2150–5. 10.1093/ndt/14.9.2150. 10.1093/ndt/14.9.2150 10489224

[26] Hitha B, Pappachan JM, Pillai HB, et al. Microalbuminuria in patients with essential hypertension and its relationship to target organ damage: an Indian experience. Saudi J Kidney Dis Transpl Off Publ Saudi Cent Organ Transpl Saudi Arab. 2008;19(3):411–9. 18445902

[27] Stempniewicz N, Vassalotti JA, Cuddeback JK, et al. Chronic kidney disease testing among primary care patients with type 2 diabetes across 24 U.S. Health care organizations. Diabetes Care. 2021;44(9):2000–9. 10.2337/dc20-2715. 34233925 10.2337/dc20-2715 PMC8740923

[28] Forsblom CM, Groop PH, Ekstrand A, Groop LC. Predictive value of microalbuminuria in patients with insulin-dependent diabetes of long duration. BMJ. 1992;305(6861):1051–3. 10.1136/bmj.305.6861.1051. 1467683 10.1136/bmj.305.6861.1051 PMC1883577

[29] Król E, Rutkowski B, Czarniak P, et al. Early detection of chronic kidney disease: results of the PolNef study. Am J Nephrol. 2009;29(3):264–73. 10.1159/000158526. 18812692 10.1159/000158526 PMC2786021

[30] Soegondo S, Prodjosudjadi W, Setiawati A. Prevalence and risk factors for microalbuminuria in a cross-sectional study of type-2 diabetic patients in indonesia: a subset of DEMAND study. Med J Indones Published Online May. 2009;1:124. 10.13181/mji.v18i2.352.

[31] Van Der Meer V, Wielders HPM, Grootendorst DC, et al. Chronic kidney disease in patients with diabetes mellitus type 2 or hypertension in general practice. Br J Gen Pract. 2010;60(581):884–90. 10.3399/bjgp10X544041. 21144198 10.3399/bjgp10X544041 PMC2991741

[32] Gouda Z, Mashaal G, Bello AK, et al. Egypt Information, Prevention, and treatment of chronic kidney disease (EGIPT-CKD) programme: prevalence and risk factors for microalbuminuria among the relatives of patients with CKD in Egypt. Saudi J Kidney Dis Transpl Off Publ Saudi Cent Organ Transpl Saudi Arab. 2011;22(5):1055–63. 21912051

[33] Chiang SC, Lee JK, Chen CH, et al. Justifying the high prevalence of microalbuminuria for type 2 diabetic patients in Taiwan with conditional probability approach–a DEMAND II study. J Chin Med Assoc JCMA. 2011;74(1):3–10. 10.1016/j.jcma.2011.01.001. 21292196 10.1016/j.jcma.2011.01.001

[34] Ležaić V, Dimković N, Peković GP, et al. Screening of a population at risk of chronic kidney disease: analysis of factors associated with low eGFR and microalbuminuria. Ren Fail. 2011;33(10):969–76. 10.3109/0886022X.2011.615969. 21929449 10.3109/0886022X.2011.615969

[35] Ong LM, Punithavathi N, Thurairatnam D, et al. Prevalence and risk factors for proteinuria: T he N ational K Idney F oundation of M Alaysia L Ifecheck H ealth S Creening programme. Nephrology. 2013;18(8):569–75. 10.1111/nep.12112. 23782264 10.1111/nep.12112

[36] Polónia J, Carvalho D, Nazaré J, et al. Renal and cardiovascular risk predictive value of two different microalbuminuria screening methods in patients with hypertension with/without diabetes in Portugal. J Hum Hypertens. 2016;30(11):726–30. 10.1038/jhh.2015.120. 26740337 10.1038/jhh.2015.120

[37] Kang YU, Bae EH, Ma SK, Kim SW. Determinants and burden of chronic kidney disease in a high-risk population in korea: results from a cross-sectional study. Korean J Intern Med. 2016;31(5):920–9. 10.3904/kjim.2014.243. 26759157 10.3904/kjim.2014.243 PMC5016270

[38] Burrows NR, Vassalotti JA, Saydah SH, et al. Identifying High-Risk individuals for chronic kidney disease: results of the CHERISH community demonstration project. Am J Nephrol. 2018;48(6):447–55. 10.1159/000495082. 30472707 10.1159/000495082 PMC6624836

[39] Lee J, Chu C, Guzman D, et al. Albuminuria testing by race and ethnicity among patients with hypertension with and without diabetes. Am J Nephrol. 2019;50(1):48–54. 10.1159/000500706. 31167180 10.1159/000500706 PMC6620121

[40] Wong LL, Kalantar-Zadeh K, Page V, Hayashida G, You AS, Rhee CM. Insights from screening a Racially and ethnically diverse population for chronic kidney disease. Am J Nephrol. 2017;45(3):200–8. 10.1159/000455389. 28125810 10.1159/000455389 PMC5334263

[41] Otieno FCF, Ogola EN, Kimando MW, Mutai K. The burden of unrecognised chronic kidney disease in patients with type 2 diabetes at a County hospital clinic in kenya: implications to care and need for screening. BMC Nephrol. 2020;21(1):73. 10.1186/s12882-020-1705-3. 32111192 10.1186/s12882-020-1705-3 PMC7048110

[42] Thomas MC. The assessment and management of albuminuria in primary care. Diabetes Res Clin Pract. 2008;80(1):83–8. 10.1016/j.diabres.2007.10.024. 18093680 10.1016/j.diabres.2007.10.024

[43] Microalbuminuria en pacientes. Adultos ambulatorios sin Control nefrológico y Con factores de Riesgo de Enfermedad renal crónica En servicios de Nefrología de Perú. Nefrología. 2012;3210.3265/Nefrologia.pre2011.Nov.10865.

[44] Van Mil D, Kieneker LM, Harms E, et al. Effectiveness of a systematic home-based albuminuria screening programme to detect chronic kidney disease in high-risk individuals in primary care (SALINE): a cross-sectional screening study. eClinicalMedicine. 2025;82:103185. 10.1016/j.eclinm.2025.103185. 40247889 10.1016/j.eclinm.2025.103185 PMC12005226

[45] American Diabetes Association Professional Practice Committee, ElSayed NA, McCoy RG, et al. 4. Comprehensive medical evaluation and assessment of comorbidities: standards of care in Diabetes—2025. Diabetes Care. 2025;48(Supplement1):S59–85. 10.2337/dc25-S004. 39651988 10.2337/dc25-S004 PMC11635044

[46] Tangri N, Peach EJ, Franzén S, Barone S, Kushner PR. Patient management and clinical outcomes associated with a recorded diagnosis of stage 3 chronic kidney disease: the REVEAL-CKD study. Adv Ther. 2023;40(6):2869–85. 10.1007/s12325-023-02482-5. 37133647 10.1007/s12325-023-02482-5 PMC10219868

[47] Cutrell S, Alhomoud IS, Mehta A, Talasaz AH, Van Tassell B, Dixon DL. ACE-Inhibitors in hypertension: A historical perspective and current insights. Curr Hypertens Rep. 2023;25(9):243–50. 10.1007/s11906-023-01248-2. 37284934 10.1007/s11906-023-01248-2

[48] Alhomoud IS, Albekery MA, Alqadi R, et al. Finerenone in diabetic kidney disease: a new frontier for slowing disease progression. Front Med. 2025;12:1580645. 10.3389/fmed.2025.1580645. 10.3389/fmed.2025.1580645 PMC12171220 40529138

[49] Alhomoud IS, Wheeler S, Dixon D. Repurposing incretin therapies: a narrative review of emerging indications across cardiometabolic, liver, kidney, neurological, psychiatric, and other systems. Can J Physiol Pharmacol. 2025;103(9):298–311. 10.1139/cjpp-2025-0038. 40367521 10.1139/cjpp-2025-0038

[50] Alsalloum MA, Albekery MA, Alhomoud IS. Management of hypertension in chronic kidney disease: current perspectives and therapeutic strategies. Front Med. 2025;12:1630160. 10.3389/fmed.2025.1630160. 10.3389/fmed.2025.1630160 PMC12605421 41234923

