# Supplementary material for: Underutilization of albuminuria screening in adults with diabetes mellitus or hypertension: a systematic review and meta-analysis
Source: BMC Nephrol. 2025 Dec 4;27:18. doi: 10.1186/s12882-025-04672-5 (PMC12781434; doi:10.1186/s12882-025-04672-5)
Supplement: Supplementary file 1 — Supplementary Material 1 [file 12882_2025_4672_MOESM1_ESM.pdf]

Search Strategy:

PubMed:

(”prevalence” OR “Frequency” OR “Commonness” OR “Currency” OR “occurrence”) AND (“testing” OR “Screening”) AND (“albuminuria” OR “total protein” OR “Urine Albumin” OR “Microalbuminuria” OR “Albumin creatinine ratio” OR “Protein creatinine ratio” OR “Macroalbuminuria”) AND (“chronic kidney disease” OR “CKD” OR “chronic nephropathy” OR “Chronic Renal Disease” OR “Renal insufficiency” OR “Renal Impairment” OR “Kidney failure” OR “Chronic renal failure” OR “end-stage kidney disease” OR “uremia” OR “irreversible kidney failure” OR “Kidney damage”) AND (“Hypertension” OR “HTN” OR “Essential hypertension” OR “High blood pressure” OR “Cardiovascular disease”) AND (“Diabetes mellitus” OR “DM” OR “Diabetes” OR “Diabetic Kidney Disease” OR “Diabetic Nephropathy”)

Web of Science:

(prevalence* OR Frequency* OR Commonness* OR Currency* OR occurrence*) AND (test* OR screen*) AND (albuminuria* OR “total protein*” OR “Urine Albumin*” OR Microalbuminuria* OR “Albumin creatinine ratio*” OR “Protein creatinine ratio*” OR Macroalbuminuria* OR ACR* OR PCR* OR proteinuria*) AND (“chronic kidney disease*” OR CKD* OR “chronic nephropathy*” OR “Chronic Renal Disease*” OR “Renal insufficiency*” OR “Renal Impairment*” OR “Kidney failure*” OR “Chronic renal failure*” OR “end-stage kidney disease*” OR uremia* OR “irreversible kidney failure*” OR “Kidney damage*” OR ESKD OR dialysis*) AND (Hypertension* OR HTN* OR “Essential hypertension*” OR “High blood pressure*” OR “Cardiovascular disease*”) AND (Diabetes* OR “Diabetes mellitus*” OR DM OR “Diabetic Kidney Disease*” OR “Diabetic Nephropathy*”)

Scopus:

(prevalence) OR (frequency) OR (commonness) OR (currency) OR (occurrence) AND (testing) OR (screening) AND (albuminuria) OR (total protein) OR (urine albumin) OR (microalbuminuria) OR (albumin creatinine ratio) OR (protein creatinine ratio) OR (macroalbuminuria) AND (chronic kidney disease) OR (CKD) OR (Chronic nephropathy) OR (chronic renal disease) OR (renal insufficiency) OR (renal impairment) OR (kidney failure) OR (chronic renal failure) OR (end stage kidney disease) OR (uremia) OR (irreversible kidney failure) OR (kidney damage) AND (hypertension) OR (HTN) OR (Essential hypertension) OR (high blood pressure) OR (cardiovascular disease) AND (diabetes mellitus) OR (DM) OR (Diabetes) OR (diabetic kidney disease) OR (diabetic nephropathy)

Google Scholar:

(prevalence AND testing AND albuminuria AND hypertension AND diabetes mellitus AND chronic kidney disease) – meta-analysis
