# Supplementary material for: Underutilization of albuminuria screening in adults with diabetes mellitus or hypertension: a systematic review and meta-analysis
Source: BMC Nephrol. 2025 Dec 4;27:18. doi: 10.1186/s12882-025-04672-5 (PMC12781434; doi:10.1186/s12882-025-04672-5)
Supplement: Supplementary file 3 — Supplementary Material 3 [file 12882_2025_4672_MOESM3_ESM.pdf]

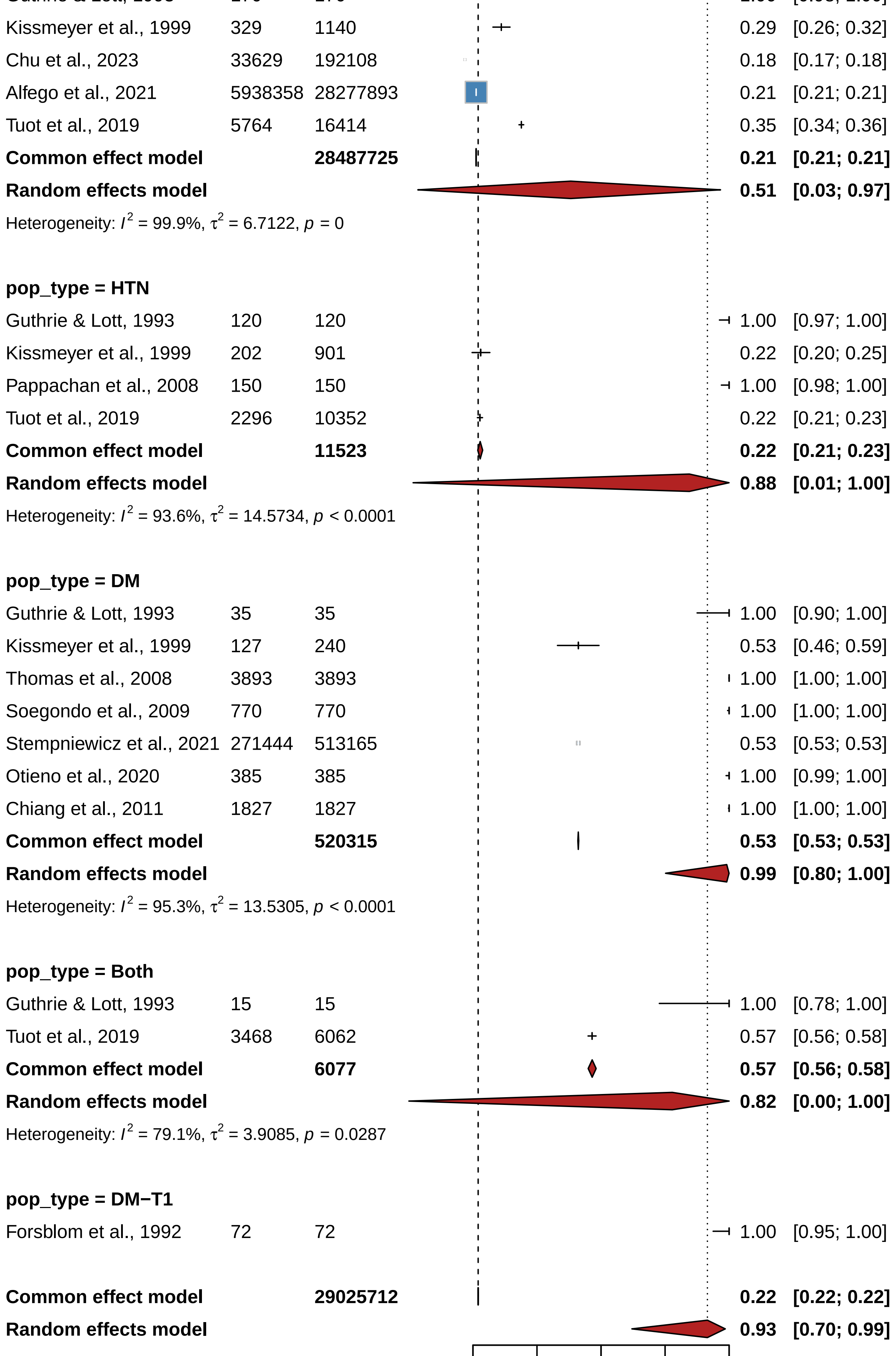
Kissmeyer et al., 1999 329 1140 0.29 [0.26; 0.32]
Chu et al., 2023 33629 192108 0.18 [0.17; 0.18]
Alfego et al., 2021 5938358 28277893 0.21 [0.21; 0.21]
Tuot et al., 2019 5764 16414 0.35 [0.34; 0.36]
Common effect model 28487725 0.21 [0.21; 0.21]
Random effects model 0.51 [0.03; 0.97]
Heterogeneity: I^2 = 99.9%, τ^2 = 6.7122, p = 0
pop_type = HTN
Guthrie & Lott, 1993 120 120 1.00 [0.97; 1.00]
Kissmeyer et al., 1999 202 901 0.22 [0.20; 0.25]
Pappachan et al., 2008 150 150 1.00 [0.98; 1.00]
Tuot et al., 2019 2296 10352 0.22 [0.21; 0.23]
Common effect model 11523 0.22 [0.21; 0.23]
Random effects model 0.88 [0.01; 1.00]
Heterogeneity: I^2 = 93.6%, τ^2 = 14.5734, p < 0.0001
pop_type = DM
Guthrie & Lott, 1993 35 35 1.00 [0.90; 1.00]
Kissmeyer et al., 1999 127 240 0.53 [0.46; 0.59]
Thomas et al., 2008 3893 3893 1.00 [1.00; 1.00]
Soegondo et al., 2009 770 770 1.00 [1.00; 1.00]
Stempniewicz et al., 2021 271444 513165 0.53 [0.53; 0.53]
Otieno et al., 2020 385 385 1.00 [0.99; 1.00]
Chiang et al., 2011 1827 1827 1.00 [1.00; 1.00]
Common effect model 520315 0.53 [0.53; 0.53]
Random effects model 0.99 [0.80; 1.00]
Heterogeneity: I^2 = 95.3%, τ^2 = 13.5305, p < 0.0001
pop_type = Both
Guthrie & Lott, 1993 15 15 1.00 [0.78; 1.00]
Tuot et al., 2019 3468 6062 0.57 [0.56; 0.58]
Common effect model 6077 0.57 [0.56; 0.58]
Random effects model 0.82 [0.00; 1.00]
Heterogeneity: I^2 = 79.1%, τ^2 = 3.9085, p = 0.0287
pop_type = DM−T1
Forsblom et al., 1992 72 72 1.00 [0.95; 1.00]
Common effect model 29025712 0.22 [0.22; 0.22]
Random effects model 0.93 [0.70; 0.99]
