# Supplementary figures and images for: Underutilization of albuminuria screening in adults with diabetes mellitus or hypertension: a systematic review and meta-analysis
Source: BMC Nephrol. 2025 Dec 4;27:18. doi: 10.1186/s12882-025-04672-5 (PMC12781434; doi:10.1186/s12882-025-04672-5)

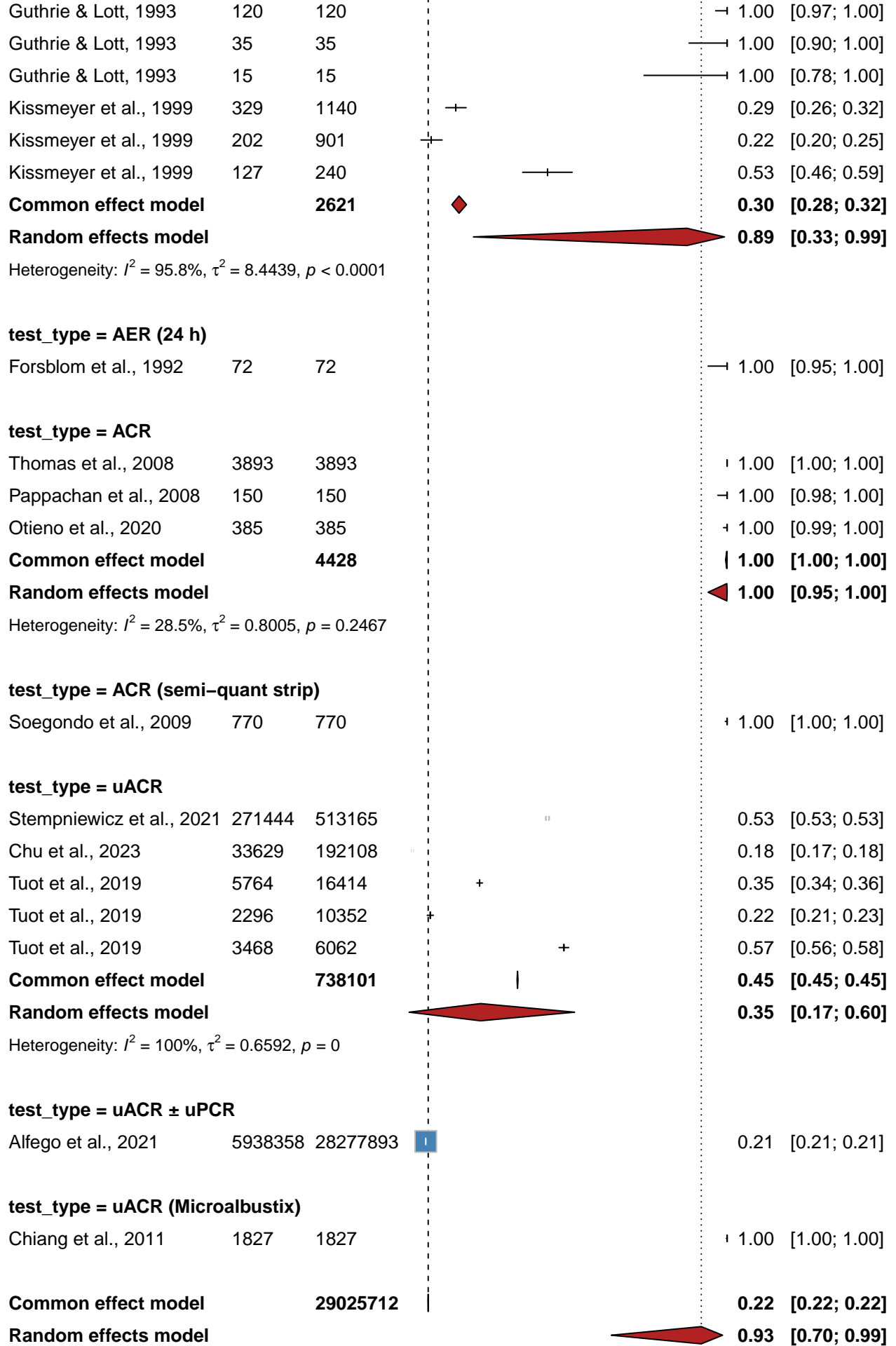

Supplement: Supplementary file 4 — Supplementary Material 4 [file 12882_2025_4672_MOESM4_ESM.pdf]
